# PARP inhibitors across different malignancies: clinical applications, resistance mechanisms, and strategies to enhance efficacy through combination therapies

**DOI:** 10.3389/fcell.2026.1891797

**Published:** 2026-09-01

**Authors:** Abdelhak Ouhajjou, Omayma Mazouji, Chakib Nejjari, Roberto Incitti, Hicham Mansour

**Affiliations:** 1 Al-Azhar Oncology Center, Rabat, Morocco; 2 GES-LCM2E, FSA, Mohamed First University, Nador, Morocco; 3 Euromed Research Center, Euromed University of Fes, Fes, Morocco; 4 Faculty of Medicine, Pharmacy, and Dentistry, Sidi Mohamed Ben Abdellah University, Fes, Morocco

**Keywords:** cancer, PARP inhibitors, precision oncology, resistance, treatment

## Abstract

Poly-ADP-ribose polymerase (PARP) inhibitors represent a novel class of anticancer agents that leverage the principle of synthetic lethality to induce tumor cell death by disrupting defective DNA repair pathways. PARP inhibitors have demonstrated strong preclinical activity in tumors with homologous recombination (HR) repair deficiencies. The discovery of synthetic lethality between HR defects and PARP inhibition led to multiple clinical trials, first in BRCA1/2-mutated cancers and later in other HR-deficient tumors. PARP inhibitors have become essential components of first- and later-line therapies for breast, prostate, pancreatic, and ovarian cancers, with several agents already approved by the FDA. However, their therapeutic potential in other malignancies remains less defined, as limited studies have explored their efficacy in additional solid tumors. This article provides an updated overview of PARP inhibitors use beyond their established clinical indications, highlighting current evidence, combination strategies, and emerging perspectives that may expand their role in various cancer treatment including endometrial cancer, cervical cancer, kidney cancer, colorectal cancer, non–small cell lung cancer (NSCLC), small cell lung cancer (SCLC) and other malignancies.

## Introduction

Poly ADP-ribose polymerase inhibitors (PARPi) have revolutionized cancer therapy, particularly in tumors with defects in homologous recombination repair (HRR) genes such as BRCA1 and BRCA2 (Lin et al., 2024). They represent one of the earliest and most prominent examples of the synthetic lethality approach in oncology describing a genetic interaction in which the loss of function of either gene alone does not impair cell viability, whereas their simultaneous inactivation leads to cell death. This loss of function may result from inactivating mutations, gene deletion, or pharmacological inhibition. PARP inhibition has been shown to initiate synthetic lethality in cells harboring BRCA1 or BRCA2 mutations, which initially supported their clinical application in some cancers. However, their therapeutic utility has expanded to a broader spectrum of malignancies exhibiting deficiencies in homologous recombination (HR) DNA repair (Muzzana et al., 2025). PARP inhibitors have received FDA approval for the treatment of breast, ovarian, pancreatic, and prostate cancers with BRCA1 and/or BRCA2 mutations. Their therapeutic efficacy is based on the principle of synthetic lethality, arising from the relationship between PARP enzymes, the base excision repair (BER) mechanism, BRCA1/BRCA2 mutations, and the homologous recombination repair (HRR) pathway. These inhibitors mainly act on PARP1 and PARP2, key enzymes responsible for repairing single-strand DNA breaks (SSBs) via the BER pathway (Akinjiyan et al., 2023; Anaeme et al., 2025).

PARP inhibitors are established as frontline treatments for breast, prostate, pancreatic, and ovarian cancers. However, their role in other malignancies remains unclear, and few studies have investigated their roles in other solid tumors. Today, PARP inhibitors are considered as a key agent in both first and later line treatments for breast, prostate, pancreatic, and ovarian cancers, with multiple PARPis approved by the Food and Drug Administration for clinical use (Alfahed, 2025; Muzzana et al., 2025). In this article, we aim to provide a comprehensive overview of the use of PARP inhibitors in the treatment of various solid tumors, extending beyond the well-established clinical indications in prostate, pancreatic, breast, and ovarian cancers. We summarize the current evidence on PARPi therapies, discuss the challenges and opportunities of combining PARPis with other treatments, and explore potential future directions and perspectives for this developed therapeutic approach.

## Methodology

This narrative review was conducted through a comprehensive literature search of PubMed and Google Scholar to identify relevant publications on the role of poly (ADP-ribose) polymerase inhibitors (PARPi) in cancer. The literature search included studies published from January 2014. Searches were performed using combinations of two or more of the following keywords: “PARP inhibitor” OR “PARPi”, “cancer”, “breast cancer”, “ovarian cancer”, “prostate cancer”, “pancreatic cancer”, “endometrial cancer”, “cervical cancer”, “non-small cell lung cancer”, “small cell lung cancer”, “mesothelioma”, “kidney cancer”, “renal cell carcinoma”, “bladder cancer”, “urothelial carcinoma”, “gastric cancer”, “colorectal cancer”, “melanoma”, “squamous cell carcinoma”, “glioblastoma”, “neuroblastoma”, “sarcoma”, “osteosarcoma”, “Ewing sarcoma”, “head and neck squamous cell carcinoma”, “hematological malignancies”, “leukemia”, “lymphoma”, “pediatric cancer”, “treatment”, “clinical trial”, “resistance mechanisms”, “predictive biomarkers”, “homologous recombination deficiency”, “DNA damage response”, and “precision oncology.”

Priority was given to clinical trials, prospective and retrospective clinical studies, high-quality preclinical investigations, systematic reviews, meta-analyses, and landmark publications evaluating the efficacy, resistance mechanisms, predictive biomarkers, and emerging combination strategies of PARP inhibitors. Additional references were identified by manually screening the bibliographies of relevant articles. Only English-language publications with sufficient methodological detail and direct relevance to the scope of this review were included. Duplicate publications, conference abstracts without full-text availability, and studies lacking relevance to PARP inhibitor therapy were excluded.

## I- PARP inhibitors

**FIGURE 1:**
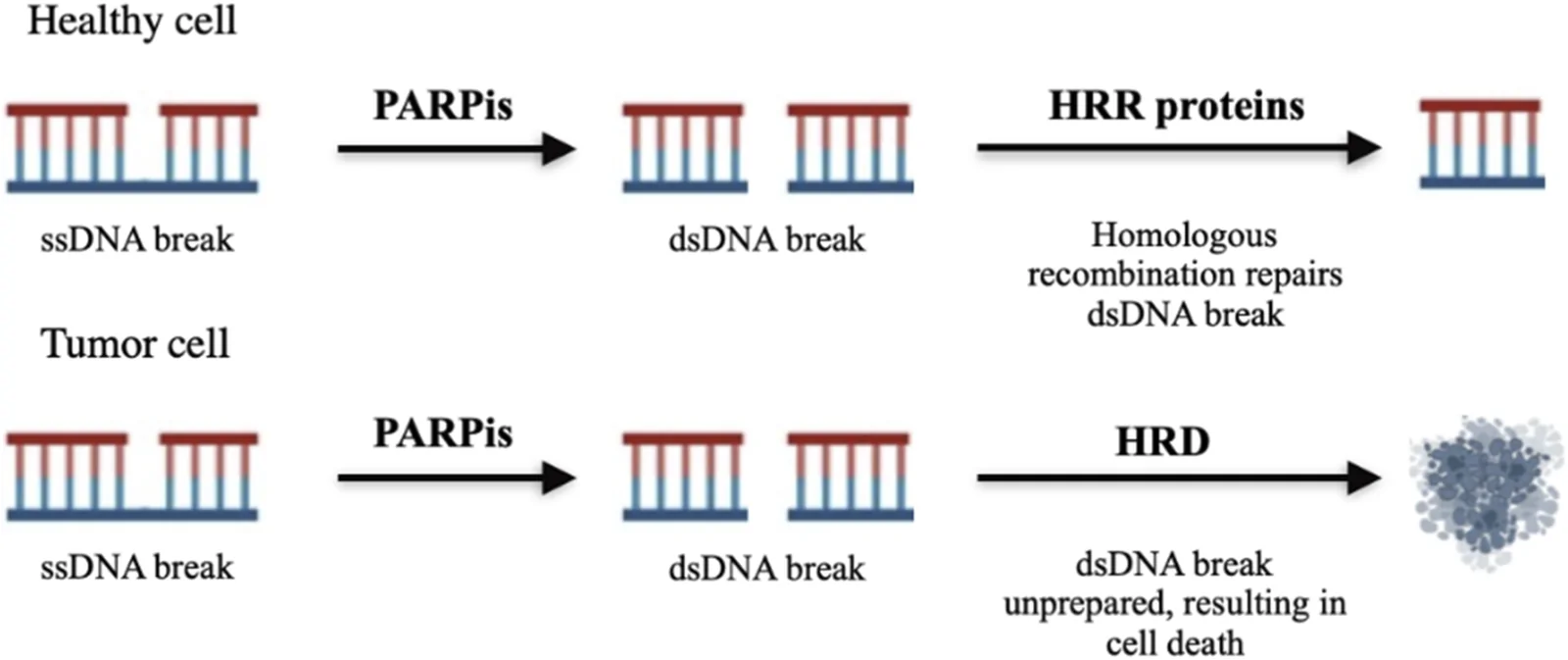
Figure demonstrating a normal cell with an intact homologous recombination repair (HRR) pathway capable of repairing double-strand DNA breaks after PARP inhibition of single-strand breaks, in contrast to HR-deficient (HRD) cells that fail to repair double-strand breaks, leading to DNA damage accumulation and subsequent cell death.

PARP (poly ADP ribose polymerase) inhibitors constitute a novel class of anticancer agents that exploit the concept of synthetic lethality, inducing tumor cell death by targeting defects in DNA repair mechanisms (Figure 1). PARPis were Initially developed and tested in patients with BRCA mutation and demonstrated efficacy across broader patient populations (Sachdev et al., 2019). PARP is a nuclear enzyme belonging to a family of 18 identified PARP proteins leading to catalyze the transfer of poly ADP-ribose (PAR) or mono-ADP-ribose units to themselves and/or other target proteins. PARPs are multifunctional enzymes involved in several key cellular processes, including cell cycle regulation, apoptosis, genomic stability, chromatin remodeling, and transcription. Among PARP proteins, PARP-1 is responsible for the majority of overall PARP activity. PARP-1 plays a central role in repairing DNA single-strand breaks (SSBs), particularly through the base excision repair (BER) pathway (Zhu et al., 2020). Defects in BER can lead to the accumulation of SSBs and their conversion into double-strand breaks (DSBs), resulting in genomic instability, which is a hallmark of cancer development and progression (Wang et al., 2025).

Furthermore, PARP-1 contributes to the activation of ATM, a key regulator of homologous recombination (HR) (Zhu et al., 2020). Homologous recombination is a key DNA repair pathway responsible for correcting double-strand breaks (DSBs) in eukaryotic cells. Defects or pathogenic mutations in genes involved in HR are strongly linked to the development of several cancers, including breast and ovarian malignancies. Cells utilize multiple DDR pathways including HR repair (HRR), base excision repair (BER), nucleotide excision repair (NER), mismatch repair (MMR), and nonhomologous end joining (NHEJ) to maintain genomic integrity (Dietlein et al., 2014). Deficiencies in HR give rise to homologous recombination deficiency (HRD), characterized by chromosomal and subchromosomal alterations including copy number variations, loss of heterozygosity (LOH), and large-scale structural rearrangements. Besides, epigenetic silencing of HR-related tumor suppressor genes through promoter hypermethylation can also induce HRD (Mekonnen et al., 2022; Wang et al., 2026).

PARP inhibitors (PARPi) represent a novel class of anticancer agents and have emerged as one of the most promising and effective classes of DNA damage response targeting agents, demonstrating efficacy both as monotherapy and in combination with other treatments to enhance tumor cell sensitivity to DNA-damaging agents (Rose et al., 2020). Clinically, the homologous recombination pathway and homologous recombination deficiency are of particular importance, as tumors harboring HRD, particularly those harboring mutations in the key HR genes BRCA1 and BRCA2 show increased susceptibility to PARP inhibition (Coleman et al., 2019; Tuli et al., 2019). Consequently, HR status serves as a critical biomarker for predicting PARPi sensitivity and therapeutic response (Mekonnen et al., 2022). Several PARP inhibitors have received regulatory approval for the treatment of BRCA-mutated ovarian, breast, and pancreatic cancers, underscoring their clinical relevance. Furthermore, as of now, other studies have investigated PARP inhibitors in various malignancies (Rose et al., 2020).

## II- landscape of PARP inhibitors in cancers

**FIGURE 2:**
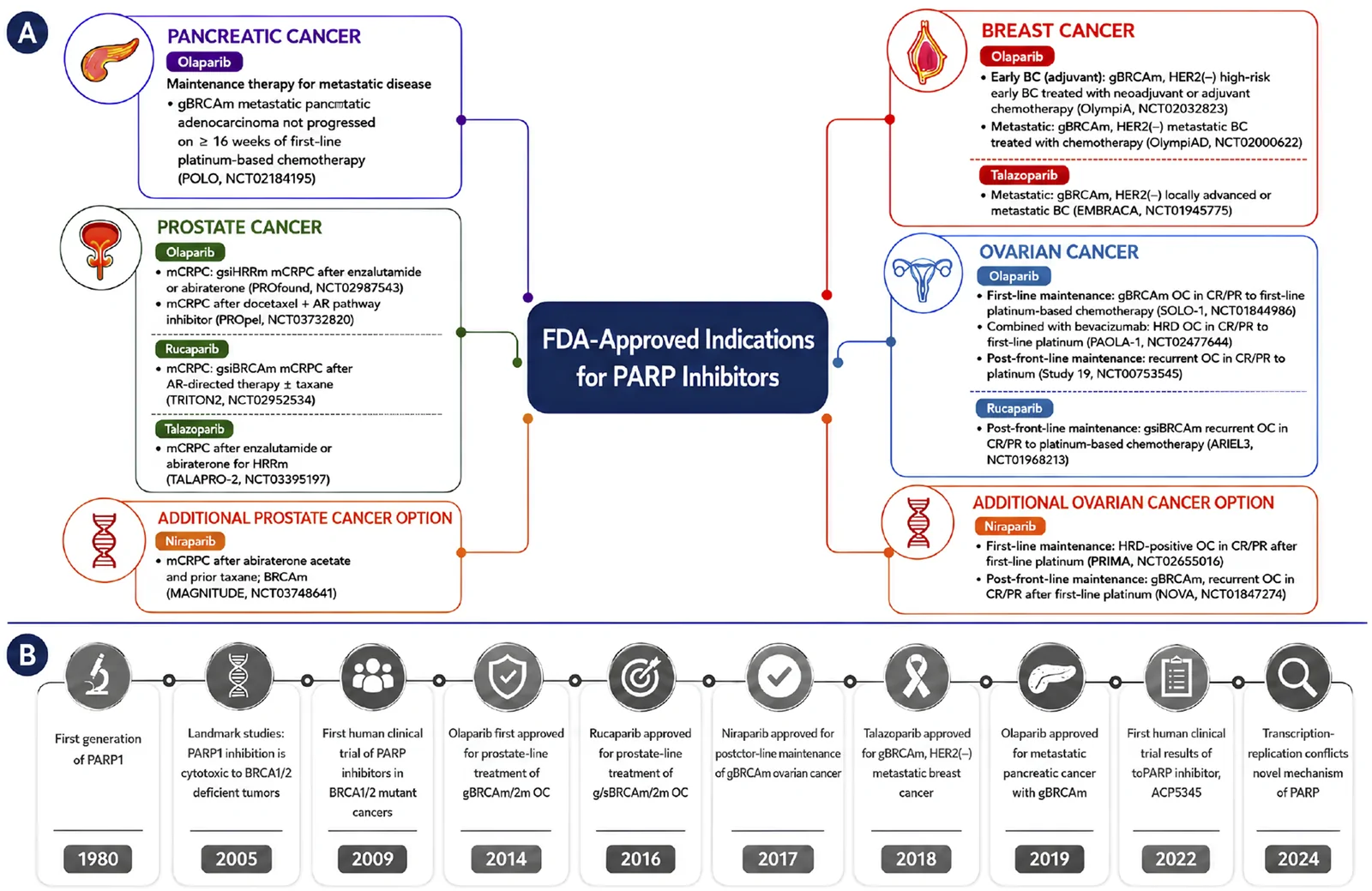
Overview of the development of PARP inhibitors (PARPi) and their FDA approvals across multiple cancers. **(A)** PARPi are approved for ovarian, breast, prostate, and pancreatic cancers, reflecting broad clinical utility. **(B)** Since the first clinical evidence of synthetic lethality in BRCA1/2-deficient patients in 2009, four PARPi have been approved for cancer treatment. The PARP1-selective inhibitor AZD5305 showed promising efficacy in early clinical trials in 2022. Beyond the classical PARP1 trapping mechanism, a new mode of action such as transcription and replication conflict was identified in 2024 (Xiang et al., 2026).

PARP inhibitors have become well-established therapy for breast, prostate, pancreatic, and ovarian cancers (Figure 2). Their efficacy in other malignancies, however, remains uncertain, as limited research has explored their use in additional solid tumors (Alfahed, 2025). In the following section, we will summarize the emerging evidence on the role of PARP inhibitors not only in breast, ovarian, pancreatic, and prostate cancers, but also in other malignancies, including endometrial cancer, cervical cancer, kidney cancer, colorectal cancer, non–small cell lung cancer (NSCLC), and small cell lung cancer (SCLC).

## II-1- approved localizations

PARP inhibitors have become an established class of targeted therapies in oncology, with several agents receiving regulatory approval across multiple cancer types. To date, four PARP inhibitors including olaparib, rucaparib, niraparib, and talazoparib have been approved by the U.S. Food and Drug Administration for selected indication in breast, ovarian, pancreatic, and prostate cancers. Their clinical use is primarily guided by validated predictive biomarkers, particularly pathogenic BRCA1/2 mutations and, in specific clinical settings, broader homologous recombination repair (HRR) deficiency. Beyond these established indications, accumulating preclinical and clinical evidence suggests that additional molecular alterations, including PALB2, ATM, RAD51C/RAD51D, BRIP1, CDK12, FANCA, CHEK2, and other DNA damage repair (DDR) genes, may influence sensitivity to PARP inhibition. However, most of these biomarkers remain investigational and require prospective clinical validation before routine implementation (Roskoski, 2025).

## In breast cancer

Breast cancer remains one of the most extensively investigated indications for poly (ADP-ribose) polymerase inhibitors (PARPi), particularly in patients harboring germline BRCA1/2 mutations and HER2-negative disease. The clinical development of PARPi has established synthetic lethality as an effective therapeutic strategy for homologous recombination-deficient tumors, leading to the approval of olaparib and talazoparib for selected patients with early and metastatic breast cancer (Zambelli et al., 2024; De et al., 2025; Shah et al., 2025; Tzang et al., 2026). Examples of clinical trials evaluating PARP inhibitors in breast cancer are summarized in Table 1.

**TABLE 1:** Examples clinical trials evaluating PARP inhibitors in breast cancer.

PARP inhibitor	Trial	Clinical setting	Main findings	Clinical significance	References
Olaparib	OlympiA	Early HER2−, gBRCA	Improved invasive DFS and distant DFS	Established standard adjuvant therapy	Tutt et al., 2021
Olaparib	OlympiAD	Metastatic HER2−, gBRCA	Improved PFS and response rate; no OS benefit	First approved PARPi in metastatic breast cancer	Robson et al., 2017
Talazoparib	EMBRACA	Advanced HER2−, gBRCA	Improved PFS, ORR and quality of life; no significant OS benefit	Alternative approved PARPi	Litton et al., 2018
Olaparib + Durvalumab	MEDIOLA	Metastatic gBRCA	Promising antitumor activity	Supports immunotherapy combinations	Domchek et al., 2020
Veliparib + Carboplatin/Paclitaxel	BROCADE3	Advanced BRCA-mutated	Improved PFS	Combination strategy under investigation	Diéras et al., 2024
Veliparib + Platinum	S1416	Metastatic TNBC	Benefit limited to BRCA-like tumors	Highlights biomarker-guided treatment	Rodler et al., 2023
Veliparib	BrighTNess	Neoadjuvant TNBC	No improvement in pCR or survival	Limited clinical role	Geyer et al., 2022
Niraparib	NCT03329937	Localized HER2− gBRCA	High radiological response and pCR	Encouraging neoadjuvant activity	Spring et al., 2022
Niraparib + Pembrolizumab	TOPACIO/KEYNOTE-162	Advanced TNBC	Higher responses in BRCA/HRD tumors	Supports biomarker-selected immunotherapy combinations	Turner et al., 2021
Fluzoparib	Ongoing phase II/III	HER2− metastatic	Trials ongoing	Emerging PARPi	Zhou and Zhang, 2025
AZD5305 (Saruparib)	Preclinical/Early clinical	HR-deficient tumors	Greater efficacy and lower hematologic toxicity than olaparib	Promising next-generation PARP1 inhibitor	Illuzzi et al., 2022; Herencia-Ropero et al., 2024

The strongest evidence supporting PARPi use originates from randomized phase III trials evaluating olaparib and talazoparib (Table 1). In early-stage HER2-negative breast cancer with germline BRCA1/2 pathogenic variants, the OlympiA trial demonstrated that adjuvant olaparib significantly improved invasive disease-free survival and distant disease-free survival compared with placebo, establishing olaparib as the standard adjuvant treatment for high-risk patients following chemotherapy (Tutt et al., 2021). Likewise, in metastatic disease, both the OlympiAD and EMBRACA trials consistently showed that olaparib and talazoparib significantly prolonged progression-free survival (PFS) and increased objective response rates compared with physician’s choice chemotherapy (Robson et al., 2017; Litton et al., 2018). Despite these consistent improvements in disease control, neither study demonstrated a statistically significant overall survival benefit, suggesting that PARPis primarily delay disease progression rather than substantially extending survival.

These findings have subsequently been reinforced by meta-analyses confirming significant improvements in PFS among patients with germline BRCA1/2-mutated metastatic breast cancer treated with PARPi. However, the absence of a consistent overall survival advantage across studies indicates that additional therapeutic strategies remain necessary to improve long-term clinical outcomes (Kunwor et al., 2023). Furthermore, although PARPi generally exhibit a favorable safety profile compared with conventional chemotherapy, hematologic toxicities, particularly anemia, continue to represent the principal dose-limiting adverse events (Litton et al., 2018; Kunwor et al., 2023).

Beyond approved monotherapy, considerable efforts have focused on combination strategies designed to enhance efficacy and overcome acquired resistance. The combination of olaparib with the immune checkpoint inhibitor durvalumab demonstrated encouraging antitumor activity in the MEDIOLA study, while niraparib combined with pembrolizumab produced improved responses in biomarker-selected triple-negative breast cancer, particularly among patients harboring BRCA mutations or homologous recombination deficiency (Domchek et al., 2020; Turner et al., 2021). Similarly, platinum-based combinations have yielded mixed results. Veliparib significantly improved progression-free survival when added to carboplatin and paclitaxel in the BROCADE3 trial; however, the BrighTNess trial failed to demonstrate additional benefit in the neoadjuvant setting, emphasizing that successful combination therapy depends on appropriate patient selection and disease context (Geyer et al., 2022; Diéras et al., 2024). These findings suggest that biomarker-driven treatment selection remains essential for maximizing the therapeutic benefit of PARP-based combinations.

Several next-generation PARP inhibitors are currently under clinical investigation (Table 1). Niraparib has demonstrated promising neoadjuvant activity and encouraging efficacy when combined with immune checkpoint blockade, although phase III studies have not yet established superiority over standard chemotherapy (Spring et al., 2022; Turner et al., 2021; Chauhan et al., 2026). Likewise, fluzoparib is undergoing evaluation in advanced HER2-negative breast cancer, while the highly selective PARP1 inhibitor saruparib (AZD5305) has shown superior antitumor activity with reduced hematologic toxicity in preclinical models compared with first-generation PARPi (Illuzzi et al., 2022; Herencia-Ropero et al., 2024; Ray and Opyrchal, 2025). Although these emerging agents remain investigational, they may expand treatment options by improving efficacy while reducing treatment-related toxicity.

Recent real-world evidence has further supported the effectiveness of PARPi outside randomized clinical trials. Retrospective studies have confirmed meaningful clinical activity among patients with homologous recombination repair gene alterations, while suggesting that treatment sequencing following CDK4/6 inhibitors may significantly influence outcomes in hormone receptor-positive disease (Peng et al., 2026; Zattarin et al., 2025). Nevertheless, these findings should be interpreted cautiously because of their retrospective design and potential selection bias, highlighting the need for prospective validation before incorporation into routine clinical practice.

Despite remarkable advances, several challenges continue to limit the long-term effectiveness of PARP inhibition. Approximately 40% of BRCA-deficient breast cancers exhibit primary resistance or eventually acquire resistance through restoration of homologous recombination repair, BRCA reversion mutations, loss of 53BP1, replication fork stabilization, reduced PARP trapping, and increased drug efflux (Daly et al., 2024). Future research should prioritize identifying predictive biomarkers beyond BRCA mutations, optimizing treatment sequencial, and developing rational combination therapies capable of delaying or overcoming resistance (Morganti et al., 2024).

## In ovarian cancer

Ovarian cancer represents the malignancy in which PARPi have achieved their greatest clinical impact, fundamentally changing the standard of care for patients with platinum-sensitive disease. Currently, olaparib, niraparib, and rucaparib have received regulatory approval as maintenance therapies following platinum-based chemotherapy, with the greatest benefit consistently observed in patients harboring BRCA1/2 mutations or homologous recombination deficiency. Nevertheless, accumulating evidence indicates that selected patients without BRCA mutations may also derive clinically meaningful benefit, expanding the therapeutic role of PARP inhibition beyond traditional biomarker-defined populations (O'Malley et al., 2023; Hirschl et al., 2024). Table 2 summarizes representative studies assessing the clinical efficacy of PARP inhibitors in ovarian cancer.

**TABLE 2:** Major clinical trials evaluating PARP inhibitors in ovarian cancer.

PARP inhibitor	Trial	Clinical setting	Main endpoint	Key findings	Clinical implication	References
Olaparib	Study 19	Platinum-sensitive recurrent BRCA-mutated OC	PFS	Significant improvement in PFS	Established maintenance therapy	Pujade-Lauraine et al., 2017
Olaparib	SOLO1	Newly diagnosed BRCA-mutated advanced OC	PFS	Markedly prolonged PFS with durable long-term benefit	Standard first-line maintenance	Moore et al., 2018
Olaparib	SOLO1 long-term follow-up	Advanced BRCA-mutated OC	OS/PFS	Sustained remission with clinically meaningful OS trend	Confirms durable benefit	DiSilvestro et al., 2023
Olaparib + Bevacizumab	PAOLA-1	Newly diagnosed advanced OC	PFS	Greatest benefit in HRD-positive tumors	Supports biomarker-guided combination therapy	Ray-Coquard et al., 2019
Niraparib	PRIMA/ENGOT-OV26/GOG-3012	First-line maintenance	PFS	Improved PFS in HRD-positive and overall populations	Expanded indication beyond BRCA mutations	González-Martín et al., 2019
Niraparib	PRIMA subgroup analyses	BRCA wild-type disease	PFS	Benefit maintained regardless of HR status	Broadened eligible patient population	Braicu et al., 2020
Niraparib + Pembrolizumab	TOPACIO/KEYNOTE-162	Recurrent OC	ORR	Encouraging activity irrespective of BRCA status	Investigational immunotherapy combination	Konstantinopoulos et al., 2019
Rucaparib	Study 10 + ARIEL2	Relapsed BRCA-mutated OC	ORR	High response rate and durable responses	Supported accelerated approval	Oza et al., 2017
Rucaparib	ARIEL3	Platinum-sensitive recurrent OC	PFS	Significant PFS improvement across all molecular subgroups	Standard maintenance therapy	Coleman et al., 2017
Rucaparib	ATHENA-MONO	First-line maintenance	PFS	Significant PFS benefit regardless of HRD status	Extended first-line maintenance option	Monk et al., 2022
Rucaparib	ATHENA-MONO long-term follow-up	First-line maintenance	Long-term efficacy	Durable benefit maintained after ∼5 years	Supports long-term clinical utility	Kristeleit et al., 2026
Talazoparib	Preclinical implant studies	BRCA-mutated OC	Tumor control	Improved survival with reduced systemic toxicity	Promising next-generation delivery strategy	Yang et al., 2023

Among currently approved agents, olaparib has generated the most mature evidence for maintenance therapy (Table 2). The SOLO1 trial established olaparib as the standard first-line maintenance treatment for newly diagnosed advanced BRCA-mutated ovarian cancer by demonstrating a profound and durable reduction in the risk of disease progression following platinum-based chemotherapy (Moore et al., 2018). Subsequent subgroup analyses confirmed that this benefit was maintained irrespective of baseline clinical risk, supporting broad applicability among BRCA-mutated patients (Colombo et al., 2020). Long-term follow-up further demonstrated sustained remission and clinically meaningful overall survival trends extending beyond 7 years, although the predefined statistical threshold for overall survival was not achieved (DiSilvestro et al., 2023). Quality-of-life analyses showed that prolonged disease control was achieved without compromising patient-reported outcomes, reinforcing the favorable therapeutic index of maintenance olaparib (Friedlander et al., 2021).

The therapeutic role of olaparib has subsequently expanded through rational combination strategies. In the PAOLA-1 trial, the addition of olaparib to bevacizumab significantly improved progression-free survival compared with bevacizumab alone, with the greatest benefit observed in patients with HRD-positive tumors, including those lacking BRCA mutations (Ray-Coquard et al., 2019). These findings illustrate that HRD status has become an important predictive biomarker beyond BRCA mutations and emphasize the increasing importance of molecular stratification in treatment selection.

Niraparib has similarly demonstrated substantial clinical benefits as maintenance therapy but differs from olaparib by providing efficacy across a broader molecular spectrum (Table 2). The PRIMA/ENGOT-OV26/GOG-3012 trial showed significant prolongation of progression-free survival in both HRD-positive patients and the overall study population, including patients with BRCA wild-type tumors (González-Martín et al., 2019; Braicu et al., 2020). Furthermore, individualized dosing strategies substantially reduced hematologic toxicity without compromising efficacy, highlighting the importance of treatment optimization to improve tolerability (Graybill et al., 2020). Subsequent analyses further demonstrated improvements in quality-adjusted progression-free survival together with reduced healthcare costs associated with individualized dosing, supporting both the clinical and economic value of personalized niraparib administration (Barretina-Ginesta et al., 2022; Graybill et al., 2025). Despite broad activity across molecular subgroups, progression-free survival remains the principal endpoint supporting niraparib use, while definitive overall survival benefit has yet to be consistently demonstrated.

The combination of PARP inhibition with immunotherapy represents another promising therapeutic strategy. The TOPACIO/KEYNOTE-162 study demonstrated encouraging activity of niraparib combined with pembrolizumab in heavily pretreated recurrent ovarian cancer regardless of BRCA or HRD status, suggesting that immune checkpoint blockade may partially overcome resistance to PARP inhibition (Konstantinopoulos et al., 2019). However, evidence remains preliminary, and larger randomized trials are required before these combinations can be incorporated into routine clinical practice. Similarly, nivolumab plus ipilimumab showed durable responses in a limited subset of patients with BRCA-mutated ovarian cancer but failed to achieve its predefined efficacy threshold, emphasizing that immunotherapy combinations remain investigational (Drescher et al., 2023).

Rucaparib has also demonstrated durable efficacy as maintenance therapy across multiple clinical settings (Table 2). Initial studies confirmed substantial antitumor activity in heavily pretreated BRCA-mutated ovarian cancer, while the ARIEL3 and ATHENA-MONO trials subsequently established significant progression-free survival benefit in platinum-sensitive recurrent disease and first-line maintenance therapy, respectively (Oza et al., 2017; Coleman et al., 2017; Monk et al., 2022). Long-term follow-up of ATHENA-MONO demonstrated sustained clinical benefit across homologous recombination-deficient and overall patient populations, supporting rucaparib as an effective maintenance strategy regardless of molecular subgroup, although maximal benefit continues to be observed in HRD-positive tumors (Kristeleit, 2022; Kristeleit et al., 2026).

These pivotal trials demonstrate that PARP inhibitors have transformed ovarian cancer management by substantially delaying disease progression and extending chemotherapy-free intervals. However, an important limitation across nearly all phase III studies is that progression-free survival remains the primary endpoint, whereas consistent improvements in overall survival have been less evident. Consequently, although PARPi have clearly altered disease management, further studies are required to determine their long-term survival impact and optimal sequencing with emerging targeted therapies.

Another major challenge is the development of acquired resistance. Restoration of homologous recombination repair through BRCA reversion mutations, replication fork stabilization, and alterations in DNA damage response pathways represent the principal mechanisms limiting long-term efficacy. Consequently, considerable attention has shifted toward next-generation PARP inhibitors and rational combination of therapies designed to overcome resistance. For example, localized intraperitoneal delivery of talazoparib using biodegradable implants has demonstrated superior tumor control with reduced systemic toxicity in preclinical BRCA-mutated ovarian cancer models, while combinations with ATR and PI3K inhibitors have shown promise for delaying resistance development (Yang et al., 2023). Future research should therefore prioritize biomarker-guided patient selection, optimization of combination strategies, and identification of predictive biomarkers beyond BRCA mutations to maximize the long-term clinical benefit of PARP inhibition.

## In prostate cancer

The introduction of PARPi has significantly changed the therapeutic landscape of metastatic castration-resistant prostate cancer (mCRPC), particularly in patients harboring defects in HRR genes. The identification of genomic alterations in BRCA1, BRCA2, ATM, PALB2, and other DNA damage repair genes has enabled the implementation of biomarker-driven precision medicine, resulting in the regulatory approval of olaparib and rucaparib for selected patients with advanced prostate cancer. Recent evidence further indicates that treatment selection is progressively evolving beyond BRCA1/2 mutations toward a broader molecular classification integrating multiple DNA damage response alterations and predictive biomarkers to optimize individualized therapeutic strategies (Longoria et al., 2024; Bourlon et al., 2024; Kawczak and Bączek, 2026; Mercinelli et al., 2026; Di Costanzo et al., 2026). Table 3 provides examples of the major clinical trials investigating PARP inhibitors in prostate cancer.

**TABLE 3:** Major clinical trials evaluating PARP inhibitors in prostate cancer.

PARP inhibitor	Trial	Clinical setting	Main endpoint	Key findings	Clinical implication	References
Olaparib	TOPARP-A	mCRPC with DDR alterations	Composite response	Identified HRR gene alterations as predictors of response	Established biomarker-driven therapy	Mateo et al., 2015
Olaparib	TOPARP-B	Biomarker-selected mCRPC	Antitumor activity	Confirmed efficacy in HRR-deficient tumors	Supported phase III development	Mateo et al., 2019
Olaparib	PROFOUND	HRR-mutated mCRPC after ARSI	rPFS, OS	Improved rPFS, response rate and OS	Established standard of care in HRR-mutated mCRPC	Sorrentino and Di Carlo, 2023
Rucaparib	TRITON2	BRCA-mutated mCRPC	ORR, PSA response	High response rates in BRCA1/2-mutated disease	Led to FDA approval	Abida et al., 2020a
Rucaparib	TRITON2 non-BRCA analysis	Non-BRCA DDR alterations	Clinical activity	Limited efficacy in ATM, CDK12 and CHEK2; better responses in PALB2 and FANCA	Importance of genomic stratification	Abida et al., 2020a
Talazoparib	TALAPRO-1	HRR-mutated mCRPC	ORR	Durable antitumor activity in heavily pretreated patients	Supported phase III evaluation	de Bono et al., 2021
Talazoparib + Enzalutamide	TALAPRO-2	First-line mCRPC	rPFS, OS	Improved survival outcomes, greatest benefit in HRR-deficient tumors	New first-line combination strategy	Agarwal et al., 2025
Niraparib	GALAHAD	DDR-deficient mCRPC	ORR	Highest efficacy in BRCA-mutated tumors	Reinforced BRCA-dependent activity	Smith et al., 2022
Niraparib + Abiraterone	MAGNITUDE	mCRPC	rPFS	Significant benefit in HRR-positive disease	Combination therapy for biomarker-selected patients	Chi et al., 2023
Veliparib + Abiraterone	NCT01576172	mCRPC	PFS	Preliminary evidence of benefit	NA	Sorrentino and Di Carlo, 2023

Among currently approved PARPi, olaparib has generated the strongest clinical evidence (Table 3). Early clinical studies, including TOPARP-A and TOPARP-B, established that responses to olaparib are largely restricted to tumors harboring alterations in homologous recombination repair genes, thereby validating these genomic alterations as predictive biomarkers of treatment response is needed (Mateo et al., 2015; Mateo et al., 2019). Subsequently, the phase III PROFOUND trial demonstrated that olaparib significantly improved radiographic progression-free survival, objective response rate, and overall survival compared with androgen receptor signaling inhibitors in patients with HRR-deficient mCRPC who had progressed after treatment with abiraterone or enzalutamide, establishing olaparib as the standard treatment for this molecularly defined population (Sorrentino and Di Carlo, 2023). These findings underscore the importance of comprehensive genomic testing before treatment initiation, as the therapeutic benefit of PARP inhibition is largely confined to patients with actionable DNA repair defects.

Similarly, rucaparib has demonstrated meaningful clinical activity in patients with metastatic castration-resistant prostate cancer harboring BRCA1/2 alterations (Table 3). The TRITON2 study reported high objective and prostate-specific antigen (PSA) response rates in heavily pretreated patients with BRCA-mutated disease, leading to its clinical approval in this setting (Abida et al., 2020a). However, subsequent analyses revealed considerably lower activity in tumors carrying DNA damage repair alterations other than BRCA alterations, including ATM, CDK12, and CHEK2, while more favorable responses were observed in selected patients with PALB2, FANCA, BRIP1, and RAD51B alterations (Abida et al., 2020b). These findings indicate that the efficacy of PARP inhibitors is highly dependent on the specific genomic alteration and that not all homologous recombination repair defects confer equivalent sensitivity to PARP inhibition. Emerging evidence suggests that integrating genomic, transcriptomic, and functional biomarkers may further improve prediction of treatment response and facilitate more precise patient stratification in clinical practice (Mercinelli et al., 2026).

Overall, evidence from the TOPARP-A, TOPARP-B, PROFOUND, and TRITON studies consistently demonstrates that the greatest clinical benefit of PARP inhibition is observed in patients with BRCA1/2-mutated mCRPC, whereas responses in tumors harboring other HRR alterations remain more heterogeneous. Recent comprehensive reviews emphasize that this variability reflects the biological diversity of DDR gene alterations and highlights the need to refine predictive biomarkers beyond BRCA status to improve patient selection and treatment outcomes (Mercinelli et al., 2026; Di Costanzo et al., 2026; de Bono et al., 2020).

Beyond approved monotherapy, combination strategies have emerged as an important approach for improving treatment outcomes (Table 3). Talazoparib combined with enzalutamide significantly improved radiographic progression-free survival and overall survival in the TALAPRO-2 trial, with the greatest benefit observed among patients with HRR-deficient tumors (Agarwal et al., 2025). Likewise, niraparib combined with abiraterone prolonged progression-free survival in patients harboring HRR gene alterations, supporting the integration of PARP inhibitors with androgen receptor signaling inhibitors in first-line treatment strategies (Chi et al., 2023). Although veliparib has also shown encouraging activity when combined with abiraterone, its clinical role remains investigational (Sorrentino and Di Carlo, 2023). These studies suggest that combining PARP inhibition with androgen receptor pathway blockade may extend the clinical benefit of PARPis beyond conventional BRCA-mutated populations, although prospective biomarker-guided validation remains necessary.

Despite these advances, several important limitations remain. Although PARP inhibitors produce durable responses in patients with BRCA1/2 mutations, clinical benefit is substantially reduced in many, non-BRCA, DNA repair defects, highlighting the need for more refined predictive biomarkers. Furthermore, acquired resistance inevitably develops during treatment through multiple mechanisms, including restoration of homologous recombination repair, BRCA reversion mutations, and overexpression of the drug efflux transporter ABCB1, resulting in cross-resistance to other systemic therapies (Lombard et al., 2019; Cahuzac et al., 2022; Loehr et al., 2023). Consequently, long-term disease control remains a major therapeutic challenge.

Given the heterogeneous efficacy observed across different genomic subgroups and the inevitable development of acquired resistance, current research has shifted from evaluating PARPi monotherapy toward biomarker-guided combination strategies designed to enhance treatment durability and extend clinical benefit beyond BRCA-mutated disease. Preclinical studies have demonstrated that combining PARP inhibitors with androgen receptor signaling inhibitors enhances antitumor activity even in BRCA-wildtype and androgen receptor-independent prostate cancer models, suggesting that continued androgen pathway suppression may restore PARPi sensitivity (Mallah et al., 2026). Similarly, inhibition of the ATR pathway has emerged as a promising strategy for overcoming acquired resistance. Resistant prostate cancer cells exhibit increased dependence on ATR-mediated replication stress signaling, and combining PARP inhibitors with ATR inhibitors restores treatment sensitivity in experimental models (Arce-Gallego et al., 2025). Future clinical trials should prioritize prospective validation of predictive biomarkers, refinement of HRR molecular classification beyond BRCA1/2 mutations, optimization of PARPi-based combination regimens, and identification of resistance mechanisms to improve long-term outcomes and expand the population of patients who may benefit from PARP inhibition.

## In pancreatic cancer

Pancreatic ductal adenocarcinoma (PDAC) remains one of the most aggressive malignancies, with limited therapeutic options and poor long-term survival. PARPi have emerged as a promising targeted strategy for a molecularly defined subset of patients harboring pathogenic alterations in HRR genes, particularly BRCA1, BRCA2, and PALB2 (Brown and Reiss, 2021). Clinical evidence supporting PARPi use in pancreatic cancer are summarized in Table 4.

**TABLE 4:** Major clinical trials evaluating PARP inhibitors in pancreatic cancer.

PARP inhibitor	Trial	Clinical setting	Main endpoint	Key findings	Clinical implication	References
Olaparib	Phase II	Previously treated gBRCA-mutated PDAC	ORR	Demonstrated meaningful activity in heavily pretreated patients	Supported further clinical development	Kaufman et al., 2015
Olaparib	POLO	Maintenance, metastatic gBRCA-mutated PDAC	PFS	Significantly prolonged PFS versus placebo	Established standard maintenance therapy	Golan et al., 2019
Olaparib	POLO QoL analysis	Maintenance therapy	NA	Maintained quality of life during treatment	Supports long-term maintenance use	Hammel et al., 2019
Olaparib	Phase II “BRCAness”	Advanced PDAC with DDR alterations	Clinical activity	Greatest benefit in tumors with confirmed DDR mutations	Supports molecular patient selection	Javle et al., 2021
Rucaparib	Phase II	BRCA1/2-mutated advanced PDAC	ORR	Durable responses with acceptable safety	Active in BRCA-mutated disease	Shroff et al., 2018
Rucaparib	Maintenance phase II	Platinum-sensitive BRCA/PALB2-mutated PDAC	PFS	Improved long-term disease control	Expands maintenance options	Reiss et al., 2021
Talazoparib	Phase I	Advanced PDAC	Safety/Clinical benefit	Acceptable tolerability and preliminary efficacy	Early clinical development	de Bono et al., 2017
Talazoparib	Phase II basket study	DDR-deficient solid tumors	Clinical benefit	Highest efficacy in BRCA-mutated tumors	Supports biomarker-guided use	Piha-Paul et al., 2024
Veliparib	Phase II	Previously treated PDAC	ORR	Limited single-agent activity	Combination strategies needed	Lowery et al., 2018
Veliparib + Cisplatin/Gemcitabine	Randomized phase II	BRCA/PALB2-mutated PDAC	OS/PFS	No added benefit; increased hematologic toxicity	Combination not recommended	O'Reilly et al., 2020
Olaparib + Pembrolizumab	POLAR	Maintenance HRD-positive PDAC	ORR/PFS	Promising activity in HRD-positive disease	Supports immunotherapy combinations	Park et al., 2026
Olaparib	APOLLO	Resected BRCA/PALB2-mutated PDAC	RFS	Evaluating adjuvant maintenance therapy	NA	NCT04858334, 2026
Niraparib	Phase II	HRD-positive PDAC	Clinical efficacy	Under evaluation	NA	NCT03601923, 2026
Veliparib + FOLFIRI	Phase II	Metastatic PDAC	OS	Under evaluation	Combination strategy	NCT02890355, 2026
Talazoparib	Phase II	DDR-deficient solid tumors	Pharmacodynamic activity	Sequential PARPi strategy under investigation	Future resistance-directed therapy	NCT04550494, 2026

Among currently available PARPi, olaparib has generated the strongest clinical evidence (Table 4). Early phase II studies demonstrated clinically meaningful antitumor activity in heavily pretreated patients with germline BRCA1/2 mutations, supporting further clinical development despite modest objective response rates (Kaufman et al., 2015). Subsequently, the landmark phase III POLO trial established maintenance olaparib as the first targeted therapy to significantly prolong progression-free survival in patients with metastatic germline BRCA1/2-mutated pancreatic cancer whose disease had not progressed following first-line platinum-based chemotherapy (Golan et al., 2019). Furthermore, patient-reported outcomes demonstrated that this progression-free survival benefit was achieved without compromising health-related quality of life, supporting maintenance olaparib as a clinically meaningful treatment option for this biomarker-selected population (Hammel et al., 2019). Nevertheless, no statistically significant overall survival advantage has been consistently demonstrated, indicating that delaying disease progression remains the principal clinical benefit of PARP inhibition in pancreatic cancer.

The efficacy of PARP inhibitors appears to depend strongly on the underlying genomic profile. Although olaparib has shown activity in tumors exhibiting a broader “BRCAness” phenotype, clinical responses are substantially greater in patients with confirmed DNA damage repair gene alterations, particularly BRCA1/2 and PALB2, whereas activity in unselected patients remains limited (Javle et al., 2021). These findings highlight the importance of comprehensive molecular profiling for identifying patients most likely to benefit from PARP inhibition.

Other PARP inhibitors have also demonstrated encouraging activity in biomarker-selected pancreatic cancer (Table 4). Rucaparib has shown durable responses, particularly when administered as maintenance therapy following platinum-based chemotherapy, with the greatest benefit observed in patients harboring BRCA2 or PALB2 alterations (Shroff et al., 2018; Reiss et al., 2021). Likewise, talazoparib demonstrated acceptable tolerability and promising antitumor activity in early-phase studies involving patients with homologous recombination-deficient tumors, although efficacy remained largely confined to BRCA-mutated cancers (de Bono et al., 2017; Piha-Paul et al., 2024). In contrast, veliparib has shown limited activity as monotherapy and failed to improve survival outcomes when combined with cisplatin and gemcitabine, despite increasing hematologic toxicity, suggesting that not all PARP inhibitors provide equivalent clinical benefit in pancreatic cancer (Lowery et al., 2018; O'Reilly et al., 2020).

Combination strategies are currently being explored to overcome resistance and extend the benefit of PARP inhibition. Early studies combining olaparib with gemcitabine demonstrated acceptable tolerability, whereas combinations with irinotecan-based regimens were limited by unacceptable toxicity (Bendell et al., 2015). More recently, combining PARP inhibition with immune checkpoint blockade has emerged as a promising approach. The phase II POLAR trial reported encouraging clinical activity for pembrolizumab plus olaparib in patients with HRD-positive pancreatic cancer, while demonstrating limited efficacy in HRD-negative tumors, reinforcing the importance of biomarker-guided patient selection for immunotherapy-based combinations (Park et al., 2026).

Recent real-world studies further support the effectiveness of PARP inhibitors in routine clinical practice. Retrospective analyses have confirmed that patients harboring BRCA or PALB2 mutations derive the greatest clinical benefit, whereas responses among individuals with, non-BRCA, HRR alterations are considerably more heterogeneous (Miao et al., 2024). However, these findings should be interpreted cautiously because retrospective studies are susceptible to selection bias and require prospective validation.

Despite these encouraging advances, several important limitations remain. Current evidence primarily supports PARPi use as maintenance therapy following platinum-based chemotherapy rather than as first-line treatment. Moreover, resistance to PARP inhibition inevitably develops through restoration of homologous recombination repair and other adaptive mechanisms, limiting long-term clinical benefit. Future research should therefore prioritize identifying predictive biomarkers beyond BRCA1/2, optimizing combination strategies with immunotherapy and other targeted agents, and determining the optimal sequencial treatment of PARP inhibitors within pancreatic cancer treatment algorithms. Ongoing clinical trials, including APOLLO, POLAR, and studies evaluating niraparib, veliparib, and talazoparib, are expected to further refine the role of PARP inhibition in biomarker-selected pancreatic cancer (Table 4) (NCT04858334, 2026; NCT03601923, 2026; NCT02890355, 2026; NCT04550494, 2026).

## II-2- other localizations

PARP inhibitors were initially developed and widely used for specific cancers such as breast, ovarian, prostate, and pancreatic tumors, particularly those associated with defects in DNA repair pathways like BRCA mutations. However, their clinical utility is not limited to these malignancies. Increasing evidence shows that PARP inhibitors can also be effective in a broader range of cancers, including certain gynecologic, thoracic, urologic, gastrointestinal, and even some skin cancers, especially when tumors exhibit homologous recombination deficiency or related molecular vulnerabilities. This expanding role highlights the importance of biomarker-driven therapy and supports the growing use of PARP inhibitors across multiple cancer types beyond their original indications.

## In gynecologic cancers

### Endometrial cancer

Endometrial cancer has emerged as a promising indication for PARPi, particularly in tumors exhibiting HRD, p53 abnormalities, or alterations in DNA damage response pathways. Although PARPi has not yet become standard therapy for endometrial cancer, increasing clinical evidence supports their evaluation either as monotherapy or in combination with targeted and immune-based therapies. Examples of clinical studies evaluating PARP inhibitors in endometrial cancer are summarized in Table 5.

**TABLE 5:** Examples of clinical trials evaluating PARP inhibitors in other cancers.

Cancer type	PARP inhibitor	Trial/Study	Clinical setting	Key findings	Clinical implication	References
Endometrial cancer	Olaparib	UTOLA	Maintenance after platinum	No significant PFS benefit overall; benefit in p53-abnormal tumors	Biomarker-guided treatment may improve efficacy	Joly et al., 2025
Endometrial cancer	PARPi + ICI	Network meta-analysis	Advanced/recurrent EC	Greatest PFS benefit in p53-abnormal/MMRp tumors	Supports PARPi-immunotherapy combinations	Villacampa et al., 2025
Endometrial cancer	Olaparib	DOMEC	NA	NA	Combination immunotherapy	NCT03951415, 2021
Endometrial cancer	Rucaparib	ENDOBARR	NA	NA	Triple targeted therapy	NCT03694262, 2025
Endometrial cancer	Niraparib	NA	NA	NA	Biomarker-driven combination	NCT03016338, 2024
Cervical cancer	Veliparib	Phase I/II	Recurrent disease	Modest responses; low PARP-1 associated with better outcomes	PARP-1 may predict response	Kunos et al., 2015
Cervical cancer	Veliparib	NCT01281852	NA	Acceptable safety; encouraging activity	Supports chemotherapy combinations	Thaker et al., 2017
Cervical cancer	Rucaparib	Phase II	NA	Activity enriched in ARID1A-mutated tumors	NA	Jackson et al., 2022
Cervical cancer	Niraparib	NA	NA	Enhanced radiosensitivity	Supports clinical translation	Xue et al., 2023
Cervical cancer	Multiple PARPis	NA	NA	NA	NA	Fernandes et al., 2024
NSCLC	Olaparib	PIN	Maintenance	Trend toward improved PFS	Potential role after platinum	Fennell et al., 2022
NSCLC	Olaparib	Phase I RT	CRT	Radiosensitization but increased toxicity	Combination optimization required	De Haan et al., 2021
NSCLC	Veliparib	Phase I CRT	NA	Feasible	Supports combination therapy	Kozono et al., 2021
NSCLC	Veliparib	Phase III	NA	No OS benefit	Biomarker selection needed	Govindan et al., 2022
NSCLC	Niraparib	JASPER	NA	Higher activity in PD-L1-high tumors	Supports PARPi + ICI	Ramalingam et al., 2022
SCLC	Veliparib	NA	Relapsed	Higher ORR, no OS benefit	SLFN11 predictive	Pietanza et al., 2018
SCLC	Veliparib	NA	NA	Modest PFS improvement	Biomarker-driven strategy	Owonikoko et al., 2019
SCLC	Olaparib	NA	Maintenance	No significant benefit	Not recommended in unselected patients	Woll et al., 2022
SCLC	Niraparib	NA	ES-SCLC	Small PFS benefit	Further validation required	Ai et al., 2021
SCLC	Talazoparib	NA	Maintenance	Benefit in SLFN11-positive disease	Precision oncology approach	Karim et al., 2025
Mesothelioma	Niraparib	Phase II	NA	Limited activity	Better biomarkers needed	George et al., 2024
Mesothelioma	Olaparib	Phase II	Pretreated	Limited activity	Combination strategies needed	Ghafoor et al., 2021
Kidney cancer	Talazoparib + DAC	NA	Clear-cell RCC models	Synergistic DNA damage, apoptosis, STING activation, tumor growth suppression	Strong biological rationale for epigenetic-PARP combination therapy	Zhou et al., 2023
Kidney cancer	Talazoparib + Avelumab	Phase II	Treatment-refractory metastatic disease	No objective responses; median PFS 3.5 months (VHL-RCC) and 1.2 months (FH/SDH/RMC)	Limited clinical efficacy despite strong preclinical rationale	Kotecha et al., 2024
Urothelial cancer	Talazoparib + PLX51107	NA	Cisplatin-sensitive and resistant models	Strong synergistic activity through enhanced DNA damage and apoptosis; biomarkers SLFN5, SLFN11, and OAS1 identified	Supports biomarker-guided combination strategies	Schmitz et al., 2026
Urothelial cancer	Olaparib	Phase II	Advanced/metastatic GU tumors with DDR alterations	Ongoing evaluation of olaparib in DDR-deficient tumors	Will clarify the role of PARP inhibition in molecularly selected urothelial cancers	NCT03375307, 2026
Urothelial cancer	Olaparib + Durvalumab	BISCAY (phase Ib)	Metastatic urothelial carcinoma	Modest clinical activity; greater responses observed in DDR-altered tumors	Suggests benefit may depend on molecular selection	NCT02546661, 2025
Urothelial cancer	Olaparib + Durvalumab	BAYOU (phase II)	Platinum-ineligible advanced urothelial carcinoma	Limited benefit overall; improved outcomes in HRR-mutated subgroup	Supports biomarker-driven combination therapy	NCT03459846, 2026
Gastric cancer	Niraparib	Phase II	HRD-positive metastatic gastroesophageal adenocarcinoma	No objective responses; limited efficacy	Monotherapy showed minimal benefit	Khalid et al., 2024
Gastric cancer	Pamiparib	PARALLEL-303 (phase II)	Maintenance after platinum chemotherapy	Modest PFS improvement without OS benefit	Limited role in unselected patients	Ciardiello et al., 2023
Gastric cancer	Olaparib + Paclitaxel	Phase II	Metastatic gastric cancer	Improved OS, particularly in ATM-low tumors	Suggested predictive value of ATM	Bang et al., 2015
Gastric cancer	Olaparib + Paclitaxel	GOLD (phase III)	Advanced gastric cancer	Failed to confirm significant survival benefit	ATM alone insufficient as biomarker	Bang et al., 2017
Gastric cancer	Olaparib + Ramucirumab	Phase I	Advanced gastric cancer	Acceptable safety; greater activity in HRR-mutated tumors	Supports further evaluation in biomarker-selected patients	Cecchini et al., 2024
Colorectal cancer	Olaparib ± Bevacizumab	LYNK-003	Maintenance metastatic CRC	NA	Evaluating maintenance strategy	Kim et al., 2021
Colorectal cancer	Olaparib	Phase III (NCT04456699)	Advanced CRC	No significant clinical benefit	Limited efficacy as monotherapy	Qureshi et al., 2025; Jiang et al., 2025
Colorectal cancer	Niraparib	Phase II (NCT03983993)	Advanced CRC	Ongoing evaluation with chemotherapy	Combination strategy under investigation	Jiang et al., 2025
Colorectal cancer	Fluzoparib + Camrelizumab	Phase II (NCT06516445)	Locally advanced rectal cancer	NA	Immunotherapy–PARPi combination	Jiang et al., 2025
Colorectal cancer	Talazoparib	Phase II (NCT05725200)	Metastatic CRC	NA	Biomarker-driven evaluation	Jiang et al., 2025
Colorectal cancer	Olaparib + Radiotherapy	NA	FBL-overexpressing CRC	Enhanced DNA damage and radiation response	Supports PARPi-radiotherapy combinations	Wen et al., 2025
Melanoma	Olaparib	NA	Metastatic HRD-positive melanoma	Durable responses observed in HRD-high tumors, including combination with nivolumab	Supports HRD as a potential predictive biomarke	Zhou X. et al., 2023
Melanoma	Olaparib + Nivolumab	NA	Heavily pretreated metastatic melanoma	Deep and complete responses reported in tumors harboring DDR alterations	Supports combination with immunotherapy	Khaddour et al., 2021a; Khaddour et al., 2021b
Melanoma	PARP inhibitors + MAPK inhibitors	NA	Refractory melanoma	Preliminary evidence of synergistic activity	Combination strategy warrants prospective evaluation	Phillipps et al., 2024
Melanoma	Multiple PARP inhibitors	NA	Advanced melanoma	Studies evaluating PARP inhibitors alone and in combination with immunotherapy or targeted therapy	Expected to define optimal combinations and biomarker-selected populations	Anaeme et al., 2025
Squamous cell carcinoma	Talazoparib	NA	NA	PARP1 inhibition induced DNA damage, cell-cycle arrest, and selective tumor growth inhibition	Supports clinical development of PARP inhibition in cSCC	Missero et al., 2025
Squamous cell carcinoma	Fluzoparib	NA	Metastatic BRCA2-mutated cSCC	Five-month progression-free survival with good tolerability	Suggests benefit in rare BRCA2-mutated cases; requires clinical validation	Sun C. et al., 2022
Glioblastoma	Olaparib + Temozolomide	Phase I (OPARATIC)	Recurrent GBM	Olaparib penetrated tumor tissue and showed encouraging 6-month PFS; intermittent dosing required because of myelosuppression	Supports combination with TMZ but highlights hematologic toxicity	Halford et al., 2017
Glioblastoma	Talazoparib	NA	EGFR-amplified GBM	EGFR-amplified tumors were highly sensitive owing to increased DNA damage and PARP trapping	EGFR amplification may serve as a predictive biomarker	Wu et al., 2020
Glioblastoma	Olaparib, Niraparib, Talazoparib, Veliparib, Pamiparib	NA	Newly diagnosed and recurrent GBM	Evaluation as monotherapy or combined with TMZ and radiotherapy	Expected to define optimal combinations and biomarker-selected populations	Bisht et al., 2022
Neuroblastoma	Olaparib, Talazoparib	NA	MYCN-amplified neuroblastoma	Induced replication stress and mitotic catastrophe; enhanced by CHK1 inhibition	Supports PARP inhibition in MYCN-driven disease	Colicchia et al., 2017
Neuroblastoma	Olaparib, Rucaparib	NA	High-risk neuroblastoma	Enhanced response to X-radiotherapy and I-MIBG through sustained DNA damage	Supports combination with radiotherapy and radiopharmaceuticals	Nile et al., 2016
Osteosarcoma	Olaparib	NA	Osteosarcoma models + radiotherapy	Olaparib enhanced radiosensitivity by increasing DNA damage, apoptosis, and G2/M arrest	Supports PARP inhibition as a radiosensitizing strategy in osteosarcoma	Dong et al., 2024
Osteosarcoma	PARP1/2 inhibitors	NA	RB1-deficient osteosarcoma models	RB1 loss conferred marked sensitivity to PARP inhibition independent of BRCA mutations	RB1 may serve as a predictive biomarker for PARPi therapy	Zoumpoulidou et al., 2021
Ewing sarcoma	Olaparib + trabectedin	NA	Cell lines and xenograft models	Combination produced synergistic antitumor activity with tumor regression	Supports future clinical evaluation of PARPi-based combination therapy	​
HNSCC	Olaparib	NA	HR-deficient and HR-proficient HNSCC models	Olaparib enhanced radiosensitivity, particularly in HR-deficient tumors	HR status may predict response to PARPi-radiotherapy combinations	Wurster et al., 2016
HNSCC	Olaparib	NA	NA	Low-dose olaparib achieved effective radiosensitization by inhibiting PAR formation	Lower doses may be sufficient when combined with radiotherapy	Verhagen et al., 2015
HNSCC	Olaparib, Veliparib, Talazoparib	NA	Early-phase clinical trials	Safety and efficacy of PARPi with RT and/or chemotherapy are under investigation	May establish future combination strategies in HNSCC.	Glorieux et al., 2017
Acute leukemia (AML/ALL)	Rucaparib	NA	Leukemia models	Rucaparib combined with 5-FU produced synergistic cytotoxicity through synthetic lethality	Supports PARPi-based chemotherapy combinations	Falzacappa et al., 2015
Leukemia/Lymphoma	Niraparib	NA	Combination with decitabine and HDAC inhibitors	Enhanced apoptosis and DNA damage with synergistic antitumor activity	Supports multi-agent therapeutic strategies	Valdez et al., 2017
FLT3-ITD AML	PARP inhibitors	NA	FLT3-mutated AML	FLT3 inhibition impaired DNA repair, sensitizing leukemia cells to PARP inhibition	FLT3-ITD may represent a predictive biomarker for PARPi therapy	Maifrede et al., 2018
Pediatric solid tumors	Olaparib + Irinotecan	AcSé-ESMART (phase I/II)	Relapsed/refractory pediatric cancers	Manageable toxicity with durable responses in selected patients, particularly ewing sarcoma	Supports further biomarker-guided clinical development	Gatz et al., 2026
Ewing sarcoma	Talazoparib + Temozolomide	​	Pediatric ewing sarcoma models	Strong synergistic antitumor activity	Promising combination for future pediatric clinical trials	Smith et al., 2015
Pediatric solid tumors	PARP inhibitors	​	Multiple pediatric solid tumors	PARP inhibition identified as a common therapeutic vulnerability; ribosome biogenesis proposed as biomarker	Supports precision medicine approaches in pediatric oncology	Keller et al., 2023

Among currently available PARPi, olaparib has generated the largest body of clinical evidence (Table 5). The phase IIb UTOLA trial demonstrated that maintenance olaparib did not significantly improve progression-free survival or overall survival in an unselected population with advanced or metastatic endometrial cancer following platinum-based chemotherapy (Joly et al., 2025). However, exploratory analyses suggested clinically meaningful benefit in molecularly selected subgroups, including tumors characterized by p53 abnormalities, chromosomal instability, or complete response to platinum chemotherapy, indicating that careful biomarker selection may be essential for optimizing PARPi efficacy.

Growing evidence suggests that PARP inhibition may be more effective when combined with immunotherapy than when administered as monotherapy. A recent network meta-analysis demonstrated that combining PARPi with immune checkpoint inhibitors significantly improved progression-free survival in patients with p53-abnormal, mismatch repair-proficient endometrial cancer, whereas little benefit was observed in p53-wild-type tumors. Conversely, immune checkpoint inhibitor monotherapy remained the preferred treatment for mismatch repair-deficient disease (Villacampa et al., 2025). These findings emphasize that the therapeutic value of PARP inhibitors is highly dependent on the molecular subtype of endometrial cancer and support personalized treatment strategies.

Several ongoing phase I and II clinical trials (NCT02208375, 2025; NCT02755844, 2025; NCT03951415, 2021; NCT03570437, 2022; NCT02684318, 2017; NCT03572478, 2021; NCT03552471, 2025; NCT03694262, 2025; NCT03617679, 2025; NCT03586661, 2025; NCT03016338, 2024; NCT03968406, 2025) are investigating olaparib, niraparib, rucaparib, and talazoparib in combination with immune checkpoint inhibitors, antiangiogenic agents, PI3K/AKT/mTOR inhibitors, chemotherapy, radiotherapy, and metabolic modulators (Table 5). These studies reflect a shift from PARPi monotherapy toward biomarker-guided combination approaches designed to enhance therapeutic efficacy and overcome intrinsic resistance.

Despite encouraging developments, several challenges remain. Clinical benefit has not yet been consistently demonstrated in unselected patient populations, and predictive biomarkers beyond BRCA mutations remain poorly defined. Future studies should therefore prioritize molecular stratification, validation of predictive biomarkers, and optimization of combination strategies integrating PARP inhibitors with immunotherapy and targeted therapies.

### Cervical cancer

Although PARP inhibitors have demonstrated remarkable success in BRCA-associated malignancies, their role in cervical cancer remains investigational. Current evidence suggests that PARPi may enhance the activity of chemotherapy and radiotherapy while providing clinical benefit in selected molecular subgroups rather than in unselected patient populations. Table 5 provides examples of the major clinical trials investigating PARP inhibitors in cervical cancer.

Initial clinical studies demonstrated that veliparib can be safely combined with topotecan or platinum-based chemotherapy in recurrent cervical cancer (Kunos et al., 2015; Thaker et al., 2017). Although objective response rates were modest, these studies suggested that tumors with low PARP-1 expression experienced longer progression-free and overall survival, indicating that PARP-1 expression may represent a potential predictive biomarker for treatment response. Similarly, combining rucaparib with bevacizumab demonstrated limited activity in the overall study population but appeared more effective in patients harboring ARID1A mutations, further supporting the importance of molecular patient selection (Jackson et al., 2022).

Beyond systemic therapy, PARP inhibition has also emerged as a potential radiosensitizing strategy. Preclinical studies demonstrated that combining niraparib with radiotherapy significantly enhanced tumor growth inhibition compared with either treatment alone by suppressing DNA repair pathways and increasing apoptosis (Xue et al., 2023). Although these findings remain preclinical, they provide a strong biological rationale for ongoing clinical evaluation of PARPi-radiotherapy combinations.

Several ongoing clinical trials are evaluating olaparib, niraparib, veliparib, rucaparib, and talazoparib, either as monotherapy or in combination with chemotherapy, radiotherapy, immune checkpoint inhibitors, or targeted therapies (Table 5) (Fernandes et al., 2024). Most of these studies enroll unselected patient populations without biomarker-based stratification, highlighting an important limitation of current clinical development.

Overall, the available evidence suggests that PARP inhibitors have acceptable safety profiles and promising biological activity in cervical cancer; however, definitive clinical efficacy has yet to be established. Future research should focus on identifying predictive biomarkers such as PARP-1 expression or ARID1A alterations, validating combination strategies with chemotherapy and radiotherapy, and determining the optimal role of PARP inhibition within precision oncology approaches for cervical cancer.

## In thoracic cancers

### Non-small cell lung cancer (NSCLC)

Although PARP inhibitors have transformed the management of BRCA-associated malignancies, their role in non-small cell lung cancer (NSCLC) remains investigational. Recent studies suggest that a subset of NSCLC characterized by HRD, BRCA1/2 alterations, or other defects in DNA damage repair pathways may derive clinical benefit from PARP inhibition (Soon et al., 2022; Wu et al., 2022). Clinical studies evaluating PARP inhibitors in NSCLC are summarized in Table 5.

Among currently available PARP inhibitors, olaparib and veliparib have generated the greatest clinical evidence in NSCLC (Table 5). Maintenance olaparib demonstrated encouraging improvements in progression-free survival in platinum-sensitive disease, although the primary endpoint was not statistically significant, suggesting that treatment benefit may be restricted to molecularly selected patients with homologous recombination deficiency (Fennell et al., 2022). Likewise, early-phase studies combining olaparib or veliparib with chemoradiotherapy demonstrated acceptable feasibility and biological activity but were associated with increased pulmonary, esophageal, and hematologic toxicities, emphasizing the need for improved treatment optimization (De Haan et al., 2021; Kozono et al., 2021; Argiris et al., 2021).

Combination strategies represent the major direction of current clinical development. While veliparib combined with platinum-based chemotherapy failed to improve overall survival in an unselected phase III population, exploratory analyses suggested that gene expression signatures such as LP52 may identify patients more likely to benefit from PARP inhibition (Govindan et al., 2022). Similarly, combining the PARP inhibitor niraparib with the immune checkpoint inhibitor pembrolizumab produced encouraging response rates, particularly among patients with high PD-L1 expression, supporting continued investigation of PARPi–immunotherapy combinations (Ramalingam et al., 2022).

Available studies indicate that PARP inhibitors have acceptable safety profiles and promising biological activity in NSCLC; however, clinically meaningful improvements in overall survival have not yet been consistently demonstrated. Moreover, treatment efficacy appears to depend on molecular characteristics including HRD status, BRCA alterations, and potentially other predictive biomarkers, indicating that patient selection remains a major challenge. Future clinical research should prioritize biomarker-driven patient selection, integration of PARP inhibitors with immune checkpoint blockade and radiotherapy, and identification of genomic predictors capable of selecting patients most likely to derive durable benefit from PARP inhibition.

### Small cell lung cancer (SCLC)

The clinical development of PARP inhibitors in small-cell lung cancer (SCLC) has primarily focused on enhancing the efficacy of chemotherapy and maintenance strategies in this highly aggressive malignancy. Clinical trials evaluating PARP inhibitors in SCLC are summarized in Table 5. Among the available agents, veliparib has been the most extensively investigated and has demonstrated modest improvements in PFS and objective response rates when combined with temozolomide or platinum-based chemotherapy. However, these benefits have not consistently translated into significant OS improvements, and treatment was associated with increased hematologic toxicity, including neutropenia and thrombocytopenia (Pietanza et al., 2018; Owonikoko et al., 2019; Byers et al., 2021). Importantly, several studies consistently identified SLFN11 expression as a promising predictive biomarker, with SLFN11-positive tumors deriving greater clinical benefit from PARP inhibition, highlighting the importance of biomarker-guided patient selection (Pietanza et al., 2018; Byers et al., 2021). Maintenance approaches using olaparib or niraparib similarly produced only modest improvements in PFS without significant OS benefit in unselected patients, suggesting that PARP inhibitor monotherapy has limited efficacy in the absence of molecular selection (Woll et al., 2022; Ai et al., 2021). More recently, combining the PARP inhibitor talazoparib with maintenance atezolizumab demonstrated improved PFS in patients with SLFN11-positive extensive-stage SCLC, although no significant survival advantage was observed, further supporting the concept that PARP inhibitors may be most effective when integrated into biomarker-driven combination strategies (Karim et al., 2025). Overall, current evidence indicates that PARP inhibitors have promising biological activity in SCLC but provide only modest clinical benefit in unselected populations. Future studies should therefore focus on validating predictive biomarkers such as SLFN11, optimizing combinations with immunotherapy and chemotherapy, and identifying molecular subgroups most likely to achieve durable clinical benefits from PARP inhibition.

### Mesothelioma

PARP inhibitors have recently emerged as a potential therapeutic strategy in mesothelioma because of the high frequency of alterations in BAP1 and other DNA damage response (DDR) genes. Examples of clinical studies evaluating PARP inhibitors in mesothelioma are summarized in Table 5. Although preclinical studies initially suggested that BAP1 deficiency might confer sensitivity to PARP inhibition, subsequent investigations demonstrated that BAP1 mutation status alone is not a reliable predictor of treatment response. Instead, therapeutic sensitivity appears to correlate more closely with biomarkers such as SLFN11 expression, whereas MGMT expression has been associated with resistance. Furthermore, combining PARP inhibitors with temozolomide enhanced antitumor activity in preclinical models, supporting the rationale for combination strategies (Rathkey et al., 2020). Clinical studies evaluating niraparib and olaparib have demonstrated limited single-agent activity, with low objective response rates and modest progression-free and overall survival, even in patients harboring BAP1 mutations (Ghafoor et al., 2021; George et al., 2024). Nevertheless, exploratory analyses suggested that selected patients with DDR alterations may derive modest clinical benefit, highlighting the importance of molecular stratification. Current evidence indicates that PARP inhibitor monotherapy has limited efficacy in mesothelioma and that BAP1 mutation alone is insufficient for patient selection. Future studies should therefore focus on validating predictive biomarkers beyond BAP1, including SLFN11 and other DDR-related alterations, while exploring rational combination strategies with chemotherapy or other targeted therapies to improve clinical outcomes.

## In urological cancers

### Kidney cancer

PARP inhibitors have emerged as potential therapeutic agents for kidney cancer by exploiting defects in DDR pathways; however, clinical evidence remains limited. Examples of preclinical and clinical studies evaluating PARP inhibitors in kidney cancer are summarized in Table 5. Preclinical studies have demonstrated that combining the DNA hypomethylating agent 5-aza-2′-deoxycytidine (DAC) with the PARP inhibitor talazoparib produces synergistic antitumor activity in clear-cell renal cell carcinoma (ccRCC), resulting in enhanced DNA damage, apoptosis, genomic instability, and activation of the STING-mediated immune response, thereby providing a strong biological rationale for combination therapy (Zhou X. et al., 2023). Nevertheless, clinical translation has been less encouraging. The combination of talazoparib and avelumab demonstrated limited antitumor activity in genomically unstable renal tumors, including VHL-altered clear-cell RCC, fumarate hydratase (FH)-deficient RCC, succinate dehydrogenase (SDH)-deficient RCC, and renal medullary carcinoma, with no objective responses observed across the evaluated cohorts (Kotecha et al., 2024). These findings suggest that although PARP inhibition is supported by a strong mechanistic rationale, its clinical efficacy remains insufficient as monotherapy. Future studies should therefore prioritize biomarker-guided patient selection and investigate rational combinations with immunotherapy and epigenetic therapies to improve therapeutic outcomes.

### Bladder urothelial cancer

PARP inhibitors have also been investigated in advanced urothelial carcinoma, particularly in tumors harboring alterations in DNA damage response pathways. Clinical studies evaluating PARP inhibitors in urothelial carcinoma are summarized in Table 5. Overall, PARP inhibitor monotherapy has demonstrated limited clinical efficacy in advanced disease, indicating that DNA repair defects alone are insufficient to predict therapeutic benefit (NCT03375307, 2026). Conversely, preclinical studies have shown that combining talazoparib with the BET inhibitor PLX51107 produces marked synergistic antitumor activity through enhanced DNA damage, replication stress, and apoptosis, while biomarkers including SLFN5, SLFN11, and OAS1 have emerged as potential predictors of treatment response beyond classical BRCA-associated alterations (Schmitz et al., 2026). Likewise, clinical trials evaluating olaparib in combination with immune checkpoint inhibitors, including the BISCAY and BAYOU studies, demonstrated only modest overall clinical benefit, although patients with DNA damage repair alterations appeared more likely to respond (NCT02546661, 2025; NCT03459846, 2026). The findings indicates that PARP inhibitors are unlikely to be effective as monotherapy in unselected urothelial carcinoma but may provide greater benefit in biomarker-selected populations receiving combination therapies. Future research should focus on validating predictive biomarkers and optimizing PARP inhibitor-based combination strategies to maximize clinical efficacy.

## In gastrointestinal cancers

### Gastric cancer

PARP inhibitors have attracted increasing interest in gastric cancer because a subset of tumors harbors alterations in HRR genes that may confer sensitivity to synthetic lethality-based therapies (Tai et al., 2025). Examples of clinical studies evaluating PARP inhibitors in gastric cancer are summarized in Table 5. However, clinical evidence has remained largely disappointing. PARP inhibitor monotherapy, including niraparib and pamiparib, demonstrated limited clinical efficacy, producing only modest PFS benefits without meaningful improvements in OS in advanced or metastatic gastric cancer (Khalid et al., 2024; Ciardiello et al., 2023). Likewise, combination strategies with chemotherapy have yielded mixed results. Although early studies suggested that olaparib plus paclitaxel could improve OS, particularly in patients with ATM-low tumors, these findings were not confirmed in the subsequent phase III GOLD trial, indicating that ATM expression alone is insufficient as a predictive biomarker for PARP inhibitor response (Bang et al., 2015; Bang et al., 2017). More recently, combinations with anti-angiogenic therapy have shown encouraging preliminary activity. In particular, olaparib plus ramucirumab demonstrated acceptable tolerability and greater clinical benefit in patients harboring HRR alterations, although randomized studies are still required to confirm these observations (Cecchini et al., 2024). The available evidence suggests that PARP inhibitors have limited activity in unselected gastric cancer populations, while selected patients with HRR-deficient tumors may derive greater benefit. Future research should therefore focus on validating predictive biomarkers beyond ATM, refining molecular selection, and optimizing combination strategies with anti-angiogenic agents, immunotherapy, or other targeted therapies.

### Colorectal cancer

The therapeutic role of PARP inhibitors in colorectal cancer (CRC) remains investigational despite increasing evidence supporting the importance of DNA damage repair alterations in selected molecular subgroups. Examples of preclinical and clinical studies evaluating PARP inhibitors in colorectal cancer are summarized in Table 5. Recent translational studies suggest that biomarkers such as microsatellite instability (MSI), TP53 wild-type status, PARP1/2 overexpression, and low large-scale state transition (LST) scores may better predict PARP inhibitor sensitivity than homologous recombination gene mutations alone (Smeby et al., 2020; Alfahed, 2025). Nevertheless, clinical studies evaluating PARP inhibitor monotherapy have demonstrated limited efficacy, highlighting the need for improved patient selection (Qureshi et al., 2025; Jiang et al., 2025). Consequently, current clinical development has shifted toward combination approaches, including PARP inhibitors with bevacizumab, immune checkpoint inhibitors, chemotherapy, and radiotherapy. Ongoing trials evaluating olaparib, talazoparib, niraparib, and fluzoparib aim to determine whether these combinations improve outcomes in metastatic or locally advanced colorectal cancer (Kim et al., 2021; Jiang et al., 2025). Preclinical evidence further indicates that PARP inhibition may enhance radiosensitivity, particularly in tumors overexpressing Fibrillarin (FBL), by impairing PARP1-mediated DNA repair following radiation-induced DNA damage (Wen et al., 2025). These findings indicate that PARP inhibitors are unlikely to play a major role as single-agent therapy in CRC but may become valuable components of biomarker-driven combination strategies. Future studies should focus on validating predictive biomarkers, identifying patients with DNA repair vulnerabilities, and integrating PARP inhibitors with immunotherapy, anti-angiogenic therapy, and radiotherapy to maximize clinical benefit.

## In skin cancers

### Melanoma

PARP inhibitors remain investigational in melanoma, although increasing evidence suggests that a subset of tumors characterized by HRD or other DDR alterations may benefit from PARP inhibition (Anaeme et al., 2025). Examples of preclinical and clinical studies evaluating PARP inhibitors in melanoma are summarized in Table 5. Clinical evidence is currently limited to case reports and small case series, which have demonstrated durable responses to olaparib, either as monotherapy or in combination with nivolumab, in metastatic melanoma patients harboring BRCA2, ATRX, TP53, NF1, and other DDR alterations (Zhou A. et al., 2023; Khaddour a et al., 2021; Khaddour b et al., 2021). Additional reports have suggested synergistic antitumor activity when PARP inhibitors are combined with MAPK pathway inhibitors in patients with refractory disease (Phillipps et al., 2024). Importantly, genomic analyses have shown that the estimated prevalence of HRD in melanoma varies considerably depending on the methodology used, highlighting the current lack of standardized biomarkers for patient selection (Zhou A. et al., 2023). Consequently, although preliminary findings support the biological rationale for PARP inhibition in molecularly selected melanoma, the available evidence remains insufficient for routine clinical implementation. Several ongoing clinical trials (NCT00516802, NCT03925350, NCT01618136, NCT03207347, NCT05983237, NCT01605162, and NCT04633902) are currently evaluating PARP inhibitors as monotherapy or in combination with immunotherapy and targeted therapies, with the aim of defining their clinical efficacy and validating predictive biomarkers for patient selection (Anaeme et al., 2025).

### Squamous cell carcinoma

The therapeutic application of PARP inhibitors in cutaneous squamous cell carcinoma (cSCC) remains at an early stage of development. Examples of preclinical and clinical studies evaluating PARP inhibitors in cSCC are summarized in Table 5. Preclinical studies have demonstrated that PARP1 is overexpressed in cSCC and cooperates with p63 to promote tumor proliferation, whereas PARP inhibition, particularly with talazoparib, induces persistent DNA damage, cell-cycle arrest, and selective inhibition of tumor growth while largely sparing normal keratinocytes (Missero et al., 2025). Clinical evidence, however, is currently limited to isolated case reports. In a patient with metastatic cSCC harboring a germline BRCA2 mutation, treatment with fluzoparib resulted in prolonged disease stabilization, supporting the potential role of PARP inhibition in carefully selected molecular subgroups (Sun X. et al., 2022). Despite these encouraging observations, prospective clinical evidence is lacking, and no dedicated interventional trials have yet established the efficacy of PARP inhibitors in cSCC. Future studies should therefore focus on biomarker-driven clinical trials and rational combination strategies to determine the clinical value of PARP inhibition in this disease.

## In brain tumors

### Glioblastoma

PARP inhibitors have emerged as promising therapeutic agents in glioblastoma (GBM) because of their ability to potentiate the effects of DNA-damaging therapies such as temozolomide (TMZ) and radiotherapy (Bisht et al., 2022). Examples of preclinical and clinical studies evaluating PARP inhibitors in glioblastoma are summarized in Table 5. Early clinical studies demonstrated that olaparib effectively penetrates GBM tissue and can be safely combined with intermittent TMZ, showing encouraging progression-free survival despite dose-limiting hematologic toxicity (Halford et al., 2017). Preclinical investigations have further identified EGFR amplification as a potential predictive biomarker of sensitivity to talazoparib, suggesting that molecular selection may improve therapeutic outcomes (Wu et al., 2020). These findings support the rationale for PARP inhibition in GBM but also highlight important limitations, including treatment-related myelosuppression and the absence of validated predictive biomarkers. Several ongoing clinical trials as follows; NCT05188508, NCT03561870, NCT03991832, NCT03212274, NCT02974621, NCT03212742, NCT04740190, NCT03581292, NCT02152982, NCT04614909, NCT04221503, NCT04715620, and NCT05076513, are evaluating olaparib, niraparib, talazoparib, veliparib, and pamiparib as monotherapy or in combination with TMZ and radiotherapy to optimize treatment efficacy and identify biomarker-selected patient populations (Bisht et al., 2022).

### Neuroblastoma

PARP inhibitors have also demonstrated promising therapeutic potential in neuroblastoma, particularly in tumors characterized by MYCN amplification and high PARP1/PARP2 expression, both of which are associated with aggressive disease and poor prognosis (Colicchia et al., 2017). Examples of preclinical studies evaluating PARP inhibitors in neuroblastoma are summarized in Table 5. Experimental studies have shown that olaparib and talazoparib induce replication stress, DNA damage, and mitotic catastrophe in MYCN-amplified neuroblastoma cells, effects that are further enhanced by CHK1 inhibition (Colicchia et al., 2017). In addition, PARP inhibitors such as olaparib and rucaparib significantly increase the efficacy of radiotherapy and I-MIBG by promoting persistent DNA damage and enhancing tumor cell radiosensitivity (Nile et al., 2016). Although these findings provide strong preclinical evidence supporting PARP inhibition in high-risk neuroblastoma, clinical validation remains lacking. Future studies should therefore focus on translating these preclinical observations into biomarker-driven clinical trials and evaluating rational combination strategies with radiotherapy and targeted agents.

## In sarcomas

PARP inhibitors have emerged as promising therapeutic agents across several sarcoma subtypes, although evidence remains largely preclinical. In osteosarcoma, PARP inhibition enhances radiosensitivity by increasing DNA damage, apoptosis, and cell-cycle arrest, suggesting a potential strategy to overcome radioresistance (Dong et al., 2024). Moreover, RB1-deficient osteosarcoma appears particularly sensitive to PARP inhibition, indicating that RB1 loss may serve as a predictive biomarker independent of homologous recombination deficiency (Zoumpoulidou et al., 2021). In Ewing sarcoma, preclinical studies have demonstrated synergistic antitumor activity when olaparib is combined with trabectedin, supporting further clinical evaluation of this strategy. These findings supports that PARP inhibitors are promising candidates for biomarker-driven and combination therapies in sarcoma; however, clinical validation is still lacking, and prospective trials are required to confirm their efficacy and identify patients most likely to benefit (Table 5).

## In head and neck cancers

PARP inhibitors are being investigated as radiosensitizing agents in head and neck squamous cell carcinoma (HNSCC), where treatment resistance is partly driven by defects in DNA damage repair pathways. Preclinical studies have consistently shown that olaparib enhances the effects of radiotherapy, particularly in homologous recombination-deficient tumors, by increasing DNA damage and chromosomal instability while allowing radiosensitization at relatively low drug concentrations (Wurster et al., 2016; Verhagen et al., 2015). These encouraging findings have led to several ongoing early-phase clinical trials evaluating olaparib, veliparib, and talazoparib as monotherapy or in combination with radiotherapy and chemotherapy (NCT02686008, NCT02229656, NCT02308072, NCT01491139, NCT02882308, NCT01758731, and NCT01711541) (Glorieux et al., 2017). Nevertheless, clinical evidence remains limited, and future studies should focus on validating predictive biomarkers and defining the optimal treatment combinations and dosing strategies (Table 5).

## In hematological malignancies

Although PARP inhibitors were initially developed for solid tumors with homologous recombination deficiency, growing evidence suggests they may also be effective in hematological malignancies characterized by defects in DNA repair pathways (Skelding and Lincz, 2021). Preclinical studies have demonstrated that PARP inhibitors enhance the activity of conventional chemotherapy and epigenetic therapies in leukemia and lymphoma by increasing DNA damage and impairing DNA repair (Falzacappa et al., 2015; Valdez et al., 2017). In addition, biomarker-driven approaches have identified FLT3-ITD-positive acute myeloid leukemia as a potential setting in which PARP inhibition may induce synthetic lethality and improve therapeutic efficacy (Maifrede et al., 2018). Despite these encouraging findings, clinical evidence remains scarce, and future studies are needed to validate predictive biomarkers and determine the role of PARP inhibitors in combination-based treatment strategies (Table 5).

## In pediatric cancers

PARP inhibitors are increasingly being explored in pediatric oncology, where genomic analyses have identified DNA damage repair pathways as promising therapeutic targets across several solid tumors, including Ewing sarcoma, neuroblastoma, osteosarcoma, medulloblastoma, and rhabdomyosarcoma (Keller et al., 2023). Early clinical evaluation in the AcSé-ESMART trial demonstrated that olaparib combined with irinotecan has manageable toxicity and encouraging activity in selected relapsed pediatric cancers, particularly Ewing sarcoma, while suggesting that genomic instability may predict treatment response (Gatz et al., 2026). Supporting preclinical evidence has shown marked synergy between talazoparib and temozolomide, especially in Ewing sarcoma models (Smith et al., 2015). Overall, PARP inhibitors represent a promising strategy for pediatric cancers, particularly when combined with conventional therapies, although larger biomarker-guided clinical trials are required before their routine clinical application (Table 5).

## III- overcoming resistance to PARP inhibitors

Targeted anticancer therapy development remains a central focus of current research, with PARP inhibitors representing a major clinical success in tumors with homologous recombination deficiency. This approach has led to the approval of multiple PARP inhibitors and the ongoing clinical evaluation of additional compounds across various cancer types. While clinical trials have shown encouraging response rates, resistance to PARP inhibition frequently emerges and limits long-term efficacy. As a result, significant research efforts are now directed toward understanding the mechanisms underlying resistance and developing strategies to overcome it (Dias et al., 2021).

## III-1- mechanisms of PARPi resistance

Resistance to PARPi is driven by multiple mechanisms that ultimately restore DNA repair capacity or reduce drug activity. Primary resistance is commonly associated with the absence of homologous recombination deficiency (HRD), emphasizing that HRD status is a more reliable predictor of response than BRCA1/2 mutations alone (Bi et al., 2024). Acquired resistance most frequently results from restoration of homologous recombination through BRCA1/2 reversion mutations, secondary alterations in other homologous recombination genes, or epigenetic reactivation of DNA repair pathways. Additional mechanisms include replication fork stabilization, increased drug efflux mediated by ABC transporters, restoration of PARylation following PARG loss, PARP1 mutations that reduce PARP trapping, and activation of compensatory signaling pathways such as Wnt/β-catenin, c-Met, Src, NF-κB, and ALK (Tobalina et al., 2021; Guo et al., 2018; Rondinelli et al., 2017; Taglialatela et al., 2017; Vaidyanathan et al., 2016; Gogola et al., 2018; Christie et al., 2019; Pettitt et al., 2018; Ma et al., 2022; Sun C. et al., 2022). Emerging evidence further implicates transcriptional regulation of homologous recombination genes, ferroptosis pathways, and post-translational modifications in PARPi resistance, highlighting the multifactorial nature of therapeutic escape (Bi et al., 2024). Collectively, these findings underscore the need for predictive biomarkers capable of identifying resistance mechanisms and guiding personalized therapeutic strategies. Detailed mechanisms are summarized in Table 6.

**TABLE 6:** Mechanisms of PARP inhibitor resistance.

Resistance mechanism	Molecular basis	Clinical significance	References
Restoration of homologous recombination	BRCA1/2 reversion mutations, PALB2/RAD51 alterations, epigenetic reactivation	Major cause of acquired resistance	Tobalina et al., 2021; Guo et al., 2018
Replication fork stabilization	Protection of stalled replication forks through PTIP, CHD4, RADX, SMARCAL1, FANCD2, SLFN11 alterations	Reduces PARPi-induced DNA damage	Rondinelli et al., 2017; Taglialatela et al., 2017
Drug efflux	ABCB1 (P-glycoprotein) overexpression	Decreases intracellular drug concentration	Vaidyanathan et al., 2016
Restoration of PARylation	Loss of PARG or ARH3	Reduces PARP trapping	Gogola et al., 2018; Christie et al., 2019
PARP1 mutations	Reduced PARP1 binding and trapping	Decreases drug efficacy	Pettitt et al., 2018
Alternative signaling activation	Wnt/β-catenin, c-Met, Src, NF-κB, ALK pathways	Promotes DNA repair and survival	Ma et al., 2022; Sun C. et al., 2022
Other adaptive mechanisms	Transcriptional regulation, ferroptosis pathways, post-translational modifications	Emerging mechanisms under investigation	Bi et al., 2024

## III-2- innovative methods to study PARPi resistance mechanisms

Accurate assessment of PARPi resistance is essential for optimizing patient selection and monitoring treatment response. Current approaches primarily rely on biomarkers of homologous recombination deficiency, including BRCA1/2 mutation testing and genomic instability assays, while next-generation sequencing, transcriptomic profiling, and multi-omics approaches are increasingly improving the identification of responsive patients (Vergote et al., 2022; Toh and Ngeow, 2021; Yuan et al., 2022). For acquired resistance, liquid biopsy technologies based on circulating tumor DNA (ctDNA) and cell-free DNA (cfDNA) have emerged as promising tools for real-time detection of resistance-associated genomic alterations (Kim et al., 2023; Bi et al., 2024). Despite these advances, standardized biomarkers for monitoring PARPi resistance remain lacking, representing an important area for future research.

## III-3 emerging strategies to overcome PARP inhibitor resistance

Multiple therapeutic strategies are being investigated to overcome PARPi resistance (Table 7). These include targeted protein degradation approaches, such as PROTACs, which eliminate PARP proteins rather than simply inhibiting their catalytic activity (Békés et al., 2022). Combination therapies involving immune checkpoint inhibitors, ATR, ATM, CHK1, or WEE1 inhibitors have demonstrated encouraging preclinical and early clinical activity by enhancing DNA damage and impairing DNA repair (Feng et al., 2017; Smith et al., 2020). Additional approaches include inhibition of drug efflux transporters, induction of ferroptosis, restoration of homologous recombination deficiency using epigenetic therapies, and the development of novel agents targeting POLθ or folate receptor-α (Bi et al., 2024; Sun et al., 2018; Xiang et al., 2026). Although several of these strategies have shown promising results, most remain under clinical investigation, and prospective biomarker-driven studies are required to determine their optimal clinical application.

**TABLE 7:** Strategies to overcome PARP inhibitor resistance.

Strategy	Mechanism	Clinical significance	References
Targeted protein degradation (PROTACs, HyT, molecular glues)	Degrades PARP proteins	May overcome resistance due to PARP mutations	Békés et al., 2022
Immune checkpoint inhibitors	Enhances antitumor immunity	Promising combination strategy	Feng et al., 2017
ATR/ATM/WEE1/CHK1 inhibitors	Inhibits DNA damage response	Restores synthetic lethality	Smith et al., 2020
Anti-angiogenic therapy	Induces HR deficiency	Proven benefit in selected tumors	Kaplan et al., 2019
PI3K/AKT/mTOR and MAPK inhibitors	Suppresses homologous recombination	Sensitizes resistant tumors	Batalini et al., 2022; Vena et al., 2018
Epigenetic therapies (HDAC, DNMT inhibitors)	NA	Restores PARPi sensitivity	Lazo, 2022
Chemotherapy and radiotherapy combinations	Increases DNA damage	Synergistic with PARPi	Kaufman et al., 2015; Li et al., 2023
Novel targeted agents (POLθ inhibitors, FRα therapies)	Exploits complementary DNA repair vulnerabilities	NA	Sun et al., 2018; Xiang et al., 2026

## III-4- feasible combination strategies to overcome PARP inhibitor resistance

Combination therapy has become one of the most promising approaches for overcoming PARPi resistance. Clinical and preclinical studies have demonstrated synergistic activity when PARPi are combined with chemotherapy, immune checkpoint inhibitors, anti-angiogenic agents, targeted therapies, epigenetic modulators, and DNA damage response inhibitors (Soung and Chung, 2023; Shoaib et al., 2025). These combinations enhance DNA damage, suppress DNA repair pathways, and remodel the tumor microenvironment, thereby restoring PARPi sensitivity. Notable examples include combinations with platinum-based chemotherapy, pembrolizumab or durvalumab, bevacizumab, PI3K/AKT/mTOR inhibitors, and ATR or WEE1 inhibitors, several of which have demonstrated encouraging efficacy in biomarker-selected populations (Kaufman et al., 2015; Li et al., 2023; Harter et al., 2023; Kaplan et al., 2019; Batalini et al., 2022; Vena et al., 2018; Lazo, 2022; Kondrashova et al., 2018). Nevertheless, increased toxicity and the lack of validated predictive biomarkers remain important challenges. Future studies should prioritize biomarker-guided combination strategies and rational sequencial treatment to maximize long-term clinical benefit.

## Conclusion

PARP inhibitors have transformed the therapeutic landscape of precision oncology, becoming an established treatment option for patients with homologous recombination repair (HRR)-deficient malignancies. While their clinical benefit is well established in breast, ovarian, pancreatic, and prostate cancers, accumulating preclinical and clinical evidence indicates that PARP inhibitors may also have therapeutic potential in a wide spectrum of malignancies, including lung, gastrointestinal, gynecological, genitourinary, skin, central nervous system, sarcoma, head and neck, hematological, and pediatric cancers. However, the efficacy observed across these tumor types remains heterogeneous, with clinical benefit generally confined to molecularly selected patient populations or achieved through combination strategies. The evidence summarized in this review highlights the central role of predictive biomarkers such as BRCA1/2 mutations, homologous recombination deficiency, and DNA damage response abnormalities in optimizing patient selection and maximizing therapeutic benefit. Nevertheless, biomarker validation and standardization remain major challenges, particularly in tumor types where HRD assays and predictive criteria are not yet well established. Another major challenge is the emergence of primary and acquired resistance to PARP inhibition. Multiple resistance mechanisms, including restoration of homologous recombination, replication fork stabilization, PARP1 alterations, drug efflux, and rewiring of DNA damage response pathways, continue to limit the durability of clinical responses. Consequently, considerable research efforts are focused on rational combination strategies involving chemotherapy, radiotherapy, anti-angiogenic agents, immune checkpoint inhibitors, epigenetic therapies, and inhibitors targeting complementary DNA repair pathways to overcome resistance and broaden the therapeutic applicability of PARP inhibitors.

Despite encouraging advances, several limitations remain. Many studies outside currently approved indications are early-phase, involve small patient cohorts, or report heterogeneous outcomes, emphasizing the need for larger biomarker-driven prospective clinical trials. Future research should prioritize the development of robust predictive biomarkers, standardized HRD assessment, improved understanding of resistance mechanisms, and optimization of combination regimens capable of producing durable clinical benefit. Overall, PARP inhibitors continue to represent one of the most important advances in targeted cancer therapy. As our understanding of DNA damage response biology, tumor heterogeneity, and mechanisms of resistance continues to evolve, PARP inhibitor-based strategies are expected to expand beyond their current indications and play an increasingly important role in precision oncology, ultimately improving outcomes for a broader population of patients.
